# Enhanced Transdermal Delivery of Bisoprolol Hemifumarate via Combined Effect of Iontophoresis and Chemical Enhancers: Ex Vivo Permeation/In Vivo Pharmacokinetic Studies

**DOI:** 10.3390/pharmaceutics13050682

**Published:** 2021-05-10

**Authors:** Mahmoud H. Teaima, Mohamed Azmi Ahmed Mohamed, Randa Tag Abd El Rehem, Saadia A. Tayel, Mohamed A. El-Nabarawi, Shahinaze A. Fouad

**Affiliations:** 1 Department of Pharmaceutics and Industrial Pharmacy, Faculty of Pharmacy, Cairo University, Cairo 11562, Egypt; randa.tag@pharma.cu.edu.eg (R.T.A.E.R.); teaima2002@yahoo.com (S.A.T.); mohamed.elnabarawi@pharma.cu.edu.eg (M.A.E.-N.); 2 Obour Pharmaceutical Industries, Cairo 13024, Egypt; mazmi_77@yahoo.com; 3 Department of Pharmaceutics and Pharmaceutical Technology, Faculty of Pharmacy, Ahram Canadian University, Giza 19228, Egypt; shahinazeamry9@gmail.com

**Keywords:** bisoprolol hemifumarate (BH), hypertension, hydrogels, ex vivo permeation, permeation enhancers, rat skin, transdermal iontophoresis, in vivo human study, Phoresor^®^ Unit II iontophoresis device

## Abstract

Bisoprolol hemifumarate (BH) is an antihypertensive drug that is used as first-line treatment for chronic hypertension and angina pectoris. Our study was performed to enhance the transdermal delivery of BH, a hydrophilic drug active with high molecular weight, through differently prepared hydrogels. The synergistic effect of permeation enhancers and iontophoresis was investigated via both ex vivo and in vivo permeation studies. Ex vivo iontophoretic permeation studies were performed by using male albino Wistar rat skin. Cellosolve^®^ hydrogel (F7) showed a 1.5-fold increase in Q_180_, Jss, and FER compared to F5 (lacking permeation enhancer). BH pharmacokinetic data were studied in human volunteers, following transdermal delivery of F7, using Phoresor^®^ Unit II iontophoresis device, compared to conventional oral tablets. F7 showed 1.9- and 2-fold higher values of C_max_ and AUC_0–40_, respectively compared to Concor^®^ tablets, as well as a smaller T_max_ (2.00 ± 2.00 h). The relative bioavailability of F7 was found to be 201.44%, relative to Concor^®^ tablets, demonstrating the significantly enhanced transdermal permeation of BH from the selected hydrogel by iontophoresis, in human volunteers. Finally, results showed the successful utility of permeation enhancers combined with iontophoresis in significantly enhanced transdermal permeation of BH, despite its large molecular weight and hydrophilic nature. Therefore, this strategy could be employed as a successful alternative route of administration to conventional oral tablets.

## 1. Introduction

Hypertension is a significant and a major risk factor for both morbidity and mortality, especially in geriatrics, which is the most rapidly growing population sector in the world. It is a chronic and asymptomatic cardiovascular disorder that is often associated with the risk of stroke, congestive heart failure, kidney failure, and dementia [1]. Collected data proved increased prevalence of hypertension with age. As a result, effective control and adherence to prescribed medications are of great importance, so as to improve elderly people’s quality of life and minimize the risk of cardiovascular complications [2].

Bisoprolol hemifumarate (BH), chemically known as 1-[4-(2-isopropoxyethoxymethyl) phenoxy]-N-isopropyl-3-aminopropan-2-ol fumarate, is a selective β1 receptor blocker [3]. It is used as a first-line treatment for hypertension, angina pectoris and congestive heart failure [4,5]. As reported by Nabarawi et al., BH has led to 46% reduction in sudden death of treated patients after almost one year of treatment [4]. Immediate release tablets are also characterized by rapid absorption and reduced hepatic first pass metabolism resulting in an oral bioavailability reaching almost 90% [3,5]. Although tablets compose the most widely used conventional oral dosage forms, their administration in multiple dosing, will in return reduce patient compliance. This will be augmented in case of elderly patients, who represent most of the populations treated from hypertension and angina pectoris. Because elderly patients always suffer from dysphagia and inability to drink water in a proper way, dealing with conventional oral tablets becomes troublesome [6]. Thus, an effective alternative is always sought to overcome this problem.

Transdermal route is considered an effective alternative to oral drug administration [7]. It helps to eliminate dysphagia encountered with elderly patients, as well as multiple dosing. Hence, it will provide great treatment impact for BH which will result in enhanced patient compliance [8]. Hydrogels are semisolid drug delivery systems that can contribute successfully to transdermal delivery of drug actives. Generally, hydrogels are composed of two main components: a gelling agent and an aqueous vehicle. They possess many favorable characteristics, such as being easily washable, spreadable, non-greasy, and biocompatible.

As a matter of fact, only few drug candidates can allow successful transdermal delivery. This could be attributed to the barrier properties of the stratum corneum (SC) which allows easy permeation of small molecules having moderate lipophilicity [9]. On the other hand, hydrophilic drug actives acquire reduced skin permeability due to reduced partitioning into skin lipid membranes [10]. BH is water soluble [11]; thus, in order to achieve successful BH transdermal hydrogel formulations, overcoming the physiological skin barrier is a major requirement. Different strategies have been used to improve drug permeation of hydrophilic drugs, including chemical permeation enhancers [10], as well as physical enhancement methods, such as iontophoresis [12].

Iontophoresis is the permeation of ionized molecules through skin membranes using an external electric current [13,14]. It can reversibly allow the transdermal permeation of charged drug molecules that are largely unable to penetrate lipophilic skin membranes. It is a non-invasive and pain-free method that can provide systemic administration while eliminating problems encountered with the oral route. Several studies reported the enhanced iontophoretic transdermal permeation of drugs, such as dexamethasone sodium phosphate [15], ketoprofen [16], and lidocaine hydrochloride [17].

Thus, the aim of the present study was to evaluate the potential of transdermal delivery of BH via iontophoresis. This involved evaluating the role of different permeation enhancers, within BH hydrogels, in improving the measured ex vivo iontophoretic parameters compared to drug solution. Moreover, BH pharmacokinetic (PK) parameters were investigated after iontophoretic transdermal application to human volunteers, in comparison with the commercially available Concor^®^ oral tablets.

## 2. Materials and Methods

### 2.1. Materials

Bisoprolol hemifumarate (BH) was supplied as a gift from Global Napi Pharmaceuticals, 6th of October, industrial zone, Cairo, Egypt. Aqualon^®^ sodium carboxymethylcellulose (7HF) (SCMC) was obtained from Hercules, Wilmington, DE, USA. Natrosol^TM^ 250 HHX Pharm hydroxyethylcellulose (HEC) was supplied from Hercules, Zwijndrecht, The Netherlands. Carbomer (Carbopol 940) (Cp940), Dermarol^TM^ 6CC and EMAROL^TM^ 80 (Tween^®^ 80) were purchased from CISME, Milan, Italy. 2-ethoxyethanol (Cellosolve^®^) was obtained from Oxfordlab, Gujarat, India. Triethanolamine was obtained from El Nasr chemical company, Cairo, Egypt. Propylene glycol was purchased from Fluka Chemie GmbH CH-9471, Buchs, Switzerland. Ethyl alcohol (95%) was purchased from El-Goumhouria chemical company, Cairo, Egypt. Distilled water was used throughout the whole study. All solvents and reagents were used as received.

### 2.2. Methods

#### 2.2.1. Determination of BH Using HPLC

A stock solution of BH in phosphate buffer (pH 7.4) was prepared to obtain a concentration of 4 mg/mL. Serial dilutions of BH in phosphate buffer (pH 7.4) containing 1.2, 1.6, 2, 2.4, and 2.8 mg/mL were prepared. HPLC procedure was performed by using ACME 9000 HPLC (YoungLin, Anyang, Korea), which was equipped with 3.9 mm × 30 cm Zobrax column packed with octyl silane chemically bonded to porous silica particles (5 µm in particle size). The mobile phase was a mixture of 20 mL triethyl amine with 1000 mL distilled water and 680 mL methyl alcohol, adjusted to pH 4. The injection loop had a volume of 50 µL. The detection wavelength (λ_max_) and the flow rate were 227 nm and 1 mL per minute (min), respectively. Sample injections were performed in triplicate, where the average peak area of the three injections were plotted against the concentration. The mean correlation coefficient (R^2^) was equal to 0.999. The procedural constant (K) was calculated from the reciprocal of the slope of the linear regression line representing the calibration curve.

#### 2.2.2. Preparation of BH Hydrogels

BH gels were prepared by using cellulose polymers as gelling agents, namely HEC and SCMC, as well as polyacrylic acid polymers, such as Cp940. BH (0.2% *w*/*w*) was dissolved in an alcoholic solution (5% *w*/*w*) under continuous stirring until a clear solution was obtained. Propylene glycol (10% *w*/*w*) was added to BH solution while stirring. It served as a humectant in all formulations [18]. Exactly weighed amounts of HEC or SCMC were added gradually with continuous stirring until no lumps were seen. Medicated solutions were kept in the refrigerator for 24 h (h), giving transparent, clear HEC and SCMC gels containing 2 mg BH per 1 g gel. Cp940 gels were prepared by using the aforementioned method, but with the addition of two drops triethanolamine at the end of preparation.

HEC BH gels were further prepared by using three different permeation enhancers, namely Dermarol^TM^ 6CC, Tween^®^ 80, and Cellosolve^®^. They were added in different concentrations to drug solutions just before gel formation. Compositions of the prepared BH gel formulations are shown in Table 1.

## 3. Evaluation of BH Gels

### 3.1. Physical Appearance

All hydrogels were examined visually for their color, clarity, and homogeneity.

### 3.2. Drug Content Determination of BH Hydrogels

Accurately weighed amount of each hydrogel was dissolved in 50 mL phosphate buffer (pH 7.4). The obtained solutions were filtered, using a 0.45 µm Millipore filter, and filtered samples were assayed for drug content, using a validated HPLC method, employing the previously obtained calibration curve equation (as previously explained under Section 2.2.1).

### 3.3. pH Determination of BH Hydrogels

One gram of each hydrogel was dispersed in 30 mL of distilled water. Then the pH was measured, using a pH meter apparatus (Jenway, Staffordshire, UK).

### 3.4. Rheological Measurements

Rheological parameters of the prepared BH hydrogels were determined using Cone and Plate Viscometer (Brookfield Co., BDV-ICP, Ringgold, GA, USA). All experiments were done at room temperature. Both ascending and descending parts of flow curves were assessed, where determinations were performed from 0.5 up to 100 revolutions per minute (rpm). Rheological behavior of all gel formulations was evaluated via plotting the shear rate values versus shear stress. Farrow’s equation was calculated in order to determine the flow behavior of the prepared gels, as follows [19]:

(1) Log D=N Log S−Log η

where “D” is the shear rate (s^−1^), “N” is the Farrow’s constant, “S” is the shear stress (dyne/cm^2^), and “η” is the viscosity in centipoises (cps). Flow is said to be dilatant (shear thickening) or plastic/pseudo-plastic (shear thinning) for “N” values smaller than or greater than one, respectively.

## 4. Preparation of Skin Membranes

Hairless albino Wistar rat skin was employed as a skin membrane model. Newly born male albino Wistar rats were sacrificed and full thickness skin was excised and inspected for any irregularities. Remaining adherent debris were removed by a scalpel. Fresh skin pieces were washed with tap water and preserved in glycerin solution (10%) at −20 °C. The study performed in this section was approved by Research Ethics Committee, Faculty of Pharmacy, Cairo University (PI number 2938, on 26 March 2021). Prior to experimentation, skin pieces were allowed to thaw at room temperature. For hydration, they were soaked in phosphate buffer (pH 7.4) for 1 h, at room temperature [20].

## 5. Custom-Made Iontophoretic Device

The desired electric current was produced from a custom-made iontophoretic device [21], that was designed in the laboratory and operated by a battery. The electric circuit acquired 220 volts (v) as an input direct electric current source. Silver wires (50 mm in length × 1 mm in diameter, 99% purity) were used to prepare silver/silver chloride electrodes; the effective length of each wire was one centimeter (cm). Electrodes were connected to the power source via crocodile copper wires. Rheostat, potentiometer and transistor were used to adjust the desired value of electric current intensity throughout the iontophoresis experiments. Steadiness of current intensity at the desired value was monitored using a Digital Multimeter (DT830, Yueqing Winston Electric Co., Ltd., Wenzhou, China) [22].

## 6. Ex Vivo Permeation Studies

**Figure 1:**
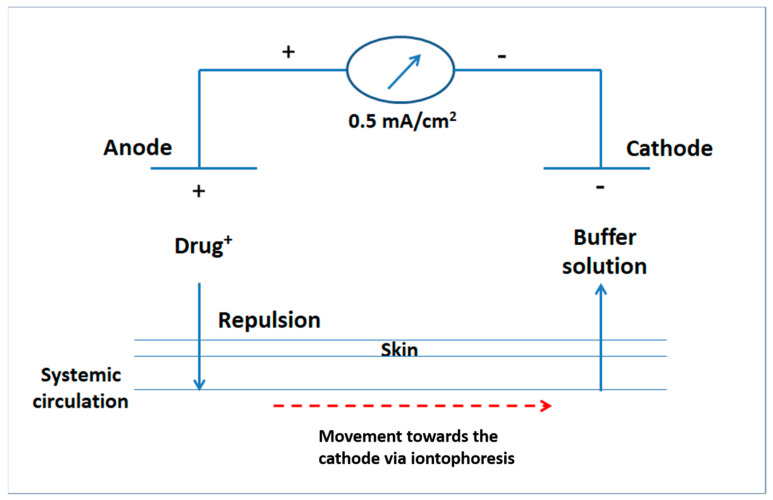
Schematic presentation of anodal iontophoresis.

Ex vivo iontophoretic transport studies were performed, using a vertical two compartment glass diffusion apparatus. It consisted of a double open sided tube. Each piece of rat skin was placed between the two compartments, where the SC faced the donor compartment (having a diameter of 2 cm) in all experiments. The surface area of skin membrane exposed to the donor solution was 3.14 cm^2^ in all cases. Experiments were carried out for a period of 3 h and extended for 1 h post-iontophoresis process. Permeation experiments were done by loading 5 mL of BH solution (2 mg/mL) or 5 mL of the selected BH gel into the donor compartment. The receptor solution contained 75 mL phosphate buffer (pH 7.4). Silver and silver chloride electrodes were employed as anode and cathode electrodes, respectively. Anodal iontophoresis (Figure 1) was performed by fixing the anodes in the donor compartment at a distance 10 mm from the rat skin. Constant direct electric current (0.5 mA/cm^2^) was applied across the rat skin. The receptor compartment was continuously stirred at 500 rpm, using Teflon-coated magnetic stirrers (3 cm) [21], in order to distribute the permeated BH solution in a homogenous way. Then, 5 mL samples were withdrawn from the receptor compartment at predetermined time intervals. Withdrawn samples were replaced with equal volumes of freshly prepared phosphate buffer (pH 7.4) in order to maintain constant volume within the receptor compartment. For the BH solution only, passive diffusion studies were simultaneously performed, using a similar setup, but without application of electric current. The cumulative amount of drug permeated was quantified via HPLC.

The mean cumulative amount of BH (Q) permeated into the receptor compartment, per unit surface area of rat’s skin (µg/cm^2^) was plotted versus time (h). The steady state flux, Jss (µg·h/cm^2^), was calculated from the slope of the linear portion of the plot [23]. Moreover, the apparent permeability coefficient (Kp) was calculated utilizing the following equation [24,25]:

Kp = Flux/Initial BH amount in the donor compartment(2)

In addition, flux enhancement ratio (FER) for BH permeation was denoted by using the following equation [23]:

FER = Jss of the selected BH gel/Jss of plain drug solution(3)

Data were statistically analyzed by one-way analysis of variance (ANOVA), followed by Tukey-Kramer test, with the significance level set at 0.05. Data were expressed as mean ± SD (Graph pad INSTAT 3.01).

## 7. Pharmacokinetic Study

### 7.1. Study Design and Subjects

The study was performed to determine the PK parameters of BH after iontophoretic delivery from the selected BH hydrogel compared to the oral delivery of the market product Concor^®^, in 5 mg tablets. The study was performed according to a blind, single-dose, two-period, random crossover design and adopted under fasting condition. Six adult, healthy, male human volunteers contributed in the study, having average weight of 76.66 ± 7.39 kg, aged between 35 and 40 years old and height from 170 to 180 cm. All volunteers were non-smokers. Their biochemical examination revealed normal liver and kidney functions. The nature and the aim of the study were completely explained to them. In addition, an informed written consent was obtained from each volunteer, where they were totally informed with the nature of the study. The clinical study protocol was reviewed and approved by the human ethical principles of Human Research Ethics Committee at the Faculty of Pharmacy, Cairo University in Egypt.

Prior to any treatment, 10 mL venous blood were withdrawn from each volunteer to measure the baseline. The study was done over two periods. In the first period, group I received the market product Concor^®^ tablets, at 5 mg (Treatment A), with 200 mL water for ingestion. Group II received the selected BH gel (Treatment B). Forearm areas of each volunteer were first swabbed with alcohol to remove any contaminants then, left to dry. The swabbed area was then marked with a circle (diameter = 2 cm). Using a syringe, 2.5 mL of the selected BH gel was applied on the marked area of the skin, using an iontophoresis device, Phoresor^®^ Unit II (Chattanooga company, Fridley, MN, USA), having TransQe iontophoresis electrodes. The active area of diffusion was 13.4 cm^2^ and the current intensity was adjusted to be 0.5 mA /cm^2^ for two hours. Both treatments were administered to fasted volunteers overnight, where only drinking water was allowed. Lunch and dinner were provided only after 4 and 10 h of drugs administration, respectively. A period of 10 days separated the two treatments as a washout period. In the second period, group I received treatment B and group II received treatment A.

### 7.2. Sample Collection

Our study was carried out under the supervision of a physician. Volunteers’ safety and samples collection were his responsibility throughout the whole study. Then, 5 mL blood samples were withdrawn from each volunteer, using indwelled cannulas at specified time intervals 0 min (pre dose), 0.5, 1, 1.5, 2, 2.5, 3, 4, 6, 9, 12, 18, 24, 36, and 40 h post-administration of each treatment. All withdrawn blood samples were filled into heparinized glass tubes, centrifuged at 3000 rpm for 10 min at 4 °C to separate plasma. Plasma was immediately stored frozen in plastic tubes at −20 °C, prior to drug analysis.

### 7.3. Sample Preparation

All samples were thawed to room temperature. A total of 50 µL of acetonitrile was added to all human plasma samples (1 mL), as the internal standard (IS). All samples were mixed for 1 min, using a vortex mixer, and then centrifuged at 3000 rpm for 10 min. The supernatant was filtered through Millipore filters (0.45 µm), and then samples were evaporated until dryness at 40 °C via centrifugal vacuum concentrator. Residues were reconstituted with the mobile phase (100 µL), where 20 µL was injected to the column via an auto sampler.

### 7.4. LC-ESI/MS Assay of BH

Plasma determination of BH was performed using a sensitive, selective and accurate liquid chromatographic method associated with electrospray ionization mass spectrometry (LC-ESI/MS), described by Ding et al. [26]. Ammonium acetate buffer (10 mM) containing 0.1% formic acid/methanol (32:68, *v/v*) was used as the mobile phase. Metoprolol was used as (IS).

### 7.5. Pharmacokinetic and Statistical Analysis

Non-compartmental analysis for determination of BH PK data, for each volunteer, was performed via Kinetica^®^ program (version 5, Thermo Fischer Scientific, New York, NY, USA). Maximum drug plasma concentrations (C_max_, ng/mL) and their corresponding time (T_max_, h) were obtained from individual plasma concentration-time curves. T_1/2_ (h) was calculated as 0.693/k. The area under the curve AUC_0–40_ (ng·h/mL), determined as the area under the plasma concentration-time curve was calculated using the linear trapezoidal rule, up to the last measured sampling time. AUC _0−∞_ (ng·h/mL) was calculated from AUC_0–40_ + Ct/K, where Ct is the last measured concentration at time (t) [27].

The obtained PK data for the selected BH hydrogel compared to that obtained from the oral treatment were analyzed for statistical significance by one-way analysis of variance (ANOVA) adopting Kinetica^®^ software. The level of significance was α = 0.05, where *p*-values less than or equal to 0.05 were considered statistically significant.

## 8. Results and Discussion

### 8.1. Physical Appearance, Drug Content and pH

All the prepared BH hydrogels exhibited homogenous texture without showing any clumps. Some of the prepared hydrogels were clear, others were turbid. Moreover, all hydrogels were colorless except F8 had a slight yellow color, which may be due to the presence of Tween^®^ 80. Mean percent of BH content ranged from 98.00 ± 0.13 to 103.00 ± 0.76 in all formulations. pH of the prepared hydrogels ranged from 7.36 ± 0.01 to 7.44 ± 0.03. All results are shown in Table 2.

### 8.2. Rheological Properties of BH Hydrogels

Viscosity of gel formulations plays a major role in drug release from their vehicles. Among the prepared hydrogels, F4 showed the lowest viscosity (645 cp) while, F6 exhibited the highest viscosity (5361 cp) (Table 3). The effect of cone speed on viscosities of the prepared hydrogels was investigated. Results showed that all hydrogels exhibited pseudo-plastic, thixotropic flow behavior that is characterized by a decrease in viscosity upon increasing the cone speed (rpm) or in case of application of high shear. In addition, rheograms of the prepared hydrogels were plotted at each point in both ascending and descending order of the cone speed (*y*-axis represented the shear rate (s^−1^) and *x*-axis represented the shear stress (dyne/cm^2^)). Hydrogels showed coincidence of the ascending and the descending curves without any hysteresis loops indicating no or negligible thixotropic breakdown. Calculated Farrow’s constant (N) for all formulations gave a value >1 (Table 3), confirming the shear thinning, non-Newtonian, pseudo plastic behavior of the prepared hydrogels. This flow behavior could be attributed to the gradual rupture of the internal structure of gels upon applying increased shear, followed by reconstruction upon shear elimination.

### 8.3. Ex Vivo Permeation of BH through Rat Skin

**Figure 2:**
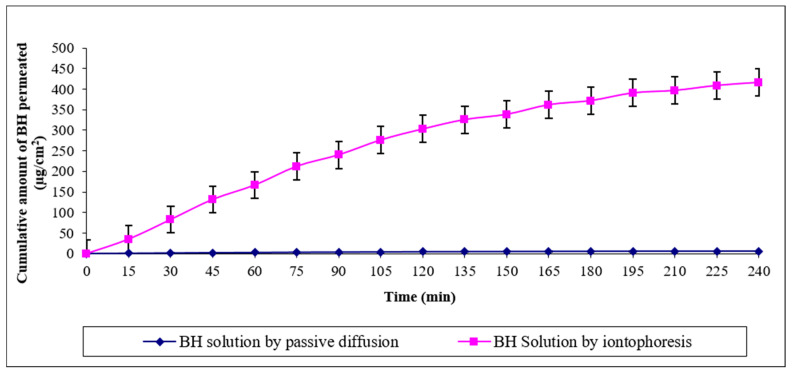
Ex vivo permeation profiles of BH solution by passive diffusion and iontophoresis through rat skin.

The measured permeation parameters of BH through rat skin are represented in Table 4. BH solution by passive diffusion showed minimal transdermal permeation (Q_180_ and Q_240_ = 5.24 ± 0.06 and 5.77 ± 0.05 µg/cm^2^), respectively, with a flux of 1.44 ± 0.01 (µg·h/cm^2^). This could be attributed to the high molecular weight of BH, where drug molecules were unable to penetrate the skin via the main routes of passive diffusion, such as trans-cellular and/or para-cellular routes. On the other hand, transdermal permeation of BH solution via iontophoresis was significantly enhanced (*p* < 0.0001) compared to passive diffusion, during the same time intervals (Figure 2), showing Q_180_ = 371.68 ± 3.78 and Q_240_ = 415.92 ± 4.44 µg/cm^2^, with a flux of 103.98 ± 1.11 µg·h/cm^2^ and permeability coefficient (kp) value of 0.01040 ± 0.00011 cm/h. Enhanced transdermal permeation of BH solution could be attributed to disarrangement of the lamellar lipid bilayer that is produced within the SC by the action of iontophoresis. Similar results were reported by Nair and Panchagnula in their study on the effect of iontophoresis on permeation of Arginine Vasopressin through rat skin [28]. Moreover, Jadoul et al. reported the potential effect of overcoming the skin’s barrier function via iontophoresis and its role in enhancing the permeation rate of drugs through the skin [29]. The applied electric field within the iontophoresis process has a major role in the enhanced transport of drug molecules through the skin. This is because electric current results in repulsion of drug molecules towards and into the skin leading to higher permeability. Similar results were obtained by Al-khalili et al. in their study on buspirone hydrochloride [30].

**Figure 3:**
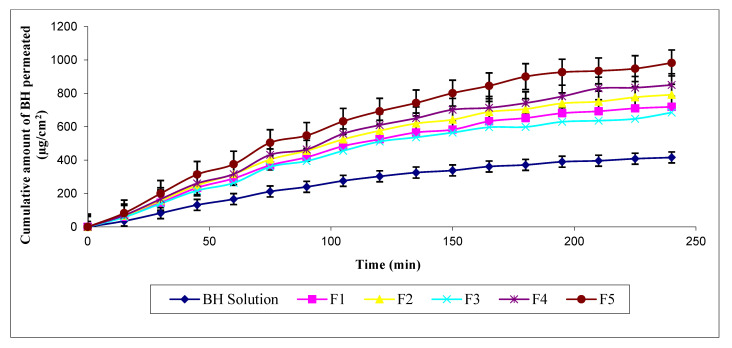
Ex vivo permeation profiles of BH hydrogels (lacking permeation enhancers) compared to BH solution by iontophoresis through rat skin.

The cumulative amount of BH permeated across rat skins after 3 h (Q_180_), from BH hydrogels (not containing permeation enhancers), can be arranged in the following ascending order: F3 (598.99 ± 5.24) < F1 (652 ± 8.06) < F2 (706.41 ± 5.14) < F4 (742.04 ± 5.67) < F5 (900.57 ± 8.79) (µg/cm^2^). Their fluxes were 171.28 ± 1.64, 179.86 ± 0.16, 198.10 ± 2.36, 212.62 ± 1.34, and 245.65 ± 1.76, µg·h/cm^2^, respectively. It is clear that iontophoretic permeation of BH was significantly enhanced from these hydrogels compared to BH solution (Figure 3). Results also showed that F5 composed of HEC as gelling agent acquired the best permeation parameters among them. It also showed the highest Q_240_ (982.61 ± 7.04 µg/cm^2^) with Kp value equivalent to (0.02457 ±0.00018). HEC is a characteristic cellulose ether derivative that is widely used for its safety and biocompatibility [31]. Several studies reported the potential of cellulose polymers for trans-mucosal transport of drugs [32,33]. They allow wetting of skin surfaces resulting in enhanced permeability. Therefore, based on the aforementioned results, HEC was chosen as the gel matrix for incorporating different hydrophilic permeation enhancers.

**Figure 4:**
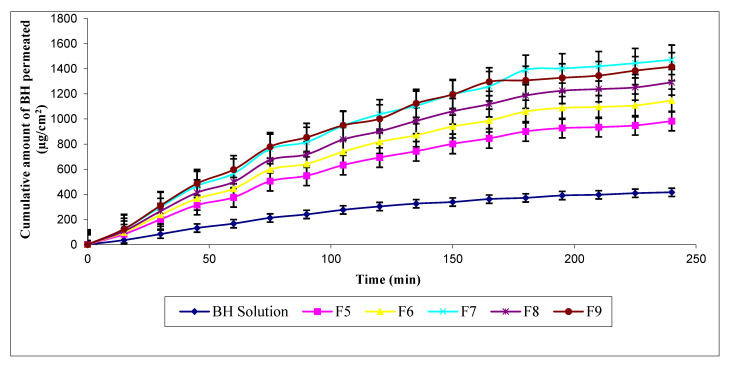
Ex vivo permeation profiles of BH hydrogels containing permeation enhancers compared to F5 (lacking permeation enhancer) and BH solution by iontophoresis through rat skin.

Results indicated significantly improved permeation pattern of BH from HEC hydrogels containing permeation enhancers, namely F6, F7, F8, and F9, compared to F5 (lacking permeation enhancer) and BH solution (Figure 4). The cumulative amount of BH permeated across rat skins after 3 h (Q_180_). These hydrogels can be arranged in the following ascending order: F6 (1058.78 ± 18.37) < F8 (1186.55 ± 12.00) < F9 (1307.44 ± 4.26) < F7 (1390.68 ± 3.65) (µg/cm^2^). Their corresponding fluxes were 286.67 ± 1.26, 323.00 ± 1.67, 353.98 ± 1.18, 367.71 ± 2.37 (µg·h/cm^2^), and their ascending kp values were 0.02867 ± 0.00013, 0.03230 ± 0.00017, 0.03540 ± 0.00012, and 0.03677 ± 0.00024 cm/h, respectively. Enhanced permeation profiles could be attributed to the addition of chemical permeation enhancers which leads to further modification in the structure of the lipid skin bilayer, resulting in widened intercellular spaces. Consequently, the transport of drug molecules via iontophoresis was effectively facilitated through the SC, where transdermal permeation was synergistically facilitated by physical and chemical enhancement techniques.

Dermarol^TM^ 6CC is chemically defined as a mixture of mono-, di-, and triglycerides of capryl/capric ethoxylates (www.cismeitaly.com, accessed on 26 March 2021). It is a derivative of PEGylated alkyl glycerides, namely polyethylene glycol-6 caprylic/capric glycerides (PEG-6-CCG). PEGylated alkyl glycerides are a sub-fraction of PEGylated oils. PEGylated oils were known to be used in cosmetics and PEGylated alkyl glycerides have been reported for their skin surfactant, as well as emulsifying properties. Its use has been reported in several research studies [34], which include a study by Kreilgaard et al. which reported the enhanced transdermal delivery of lidocaine and prilocaine hydrochloride [35]. Moreover, Gannu et al. reported the enhanced in vitro permeation of carvedilol through porcine skin owing to the action of penetration enhancers as PEGylated derivatives [36]. In addition, a study reported by Shah et al. proved the enhanced skin permeation of kahalalide F via chemical enhancers [37]. To the best of our knowledge, the literature lacks data about using PEG-6-CCG for enhancing transdermal permeation of BH. In our study, incorporating PEG-6-CCG within F6 resulted in 1.2-fold increase in Q_180_, Jss and ER compared to plain HEC hydrogel (F5). This could be attributed to the skin surfactant properties of PEG-6-CCG, as previously mentioned. Being a non-ionic surfactant, enhanced permeation is because it fluidizes lipids of the SC on two steps: first, by penetrating through the intercellular regions and solubilizing its lipid content, and second, by binding to keratin within the intercellular matrix, producing disruption within corneocytes [38]. Thus, the aforementioned sequence resulted in facilitated transport of BH molecules from F6.

Results also showed that F8 exhibited 1.3-fold increase in Q_180_, Jss and ER compared to plain HEC hydrogel (F5). This could be attributed to the skin surfactant properties of Tween^®^ 80. Being a non-ionic surfactant, its mechanism of action would be similar to PEG-6-CCG, as previously discussed. Moreover, an additional mechanism for the permeation enhancing properties of Tween^®^ 80 could be attributed to its chemical structure, containing ethylene oxide group, which helps in permeating hydrophilic drugs, such as BH. This can be confirmed by the fact of being a hydrophilic, water soluble surfactant, having high hydrophilic/lipophilic balance (HLB) value, equal to 15. This is in addition to its ability to emulsify sebum, allowing higher power of drug penetration into the skin as previously mentioned. Similar results were obtained by Nabarawi et al. in their study on transdermal delivery of BH, where Tween^®^ 80 changed the skin’s barrier properties [4], allowing for enhanced transdermal permeation. It is worth to note that Tween^®^ 80 is among the widely used and pharmaceutically acceptable permeation enhancers used for transdermal delivery of a wide variety of drug actives.

In addition, Cellosolve^®^ hydrogels, namely F7 and F9, significantly enhanced skin permeation of BH through hairless rat skin. Compared to plain HEC hydrogel (F5), F7 exhibited a 1.5-fold increase in Q_180_, Jss, and ER and F9 showed a 1.5-fold increase in Q_180_, as well as a 1.4-fold increase in Jss and ER. They also exhibited higher permeation compared to F6 and F8, as well as significantly enhanced permeation parameters compared to BH solution (*p* < 0.05).

Cellosolve^®^ is the ethylene glycol monoethyl ether, that is often known as 2-ethoxyethanol. It has been used in cosmetic preparations, such as skin lotions and oral care and nail products, as well as cleansing preparations. Moreover, it has been reported for its rapid percutaneous absorption [39]. This could be attributed to its chemical structure [40], being a derivative of glycol ethers. Glycol ether derivatives have been widely used as permeation enhancers in skin formulations. These include, for example, the use of diethylene glycol monoethyl ether (Transcutol^®^) in enhanced ex vivo transdermal permeation of each of clonazepam through rabbit ear skin [41], diclofenac through pig skin [42], and baicalin through rat skin [43].

The literature lacks data about enhanced BH skin permeation using Cellosolve^®^. Referring to 2-ethoxyethanol, as an imparted moiety within Transcutol^®^ chemical structure [44], clarifies permeation enhancement properties of Cellosolve^®^. Similarly, enhanced transdermal permeation of Cellosolve^®^ could be attributed to improving the fluidity of skin bilayers resulting in reduced skin barrier and facilitated pathway of BH towards the skin. In our study, it is worth to say that enhanced transdermal permeation was achieved by using Cellosolve^®^ with a concentration that is less three- and two-fold than its maximum allowable dose per day, within F7 and F9, respectively. It was also noted that F9 exhibited decreased values of permeation parameters, however non-significant, compared to F7. This could be attributed to its higher viscosity (981 cps) (owing to the higher Cellosolve^®^ concentration), which consequently lowered drug permeation. These results cohere with the previously performed rheological measurements. Thus, F7 was selected for further in vivo studies.

## 9. Pharmacokinetic Study in Healthy Human Volunteers

**Figure 5:**
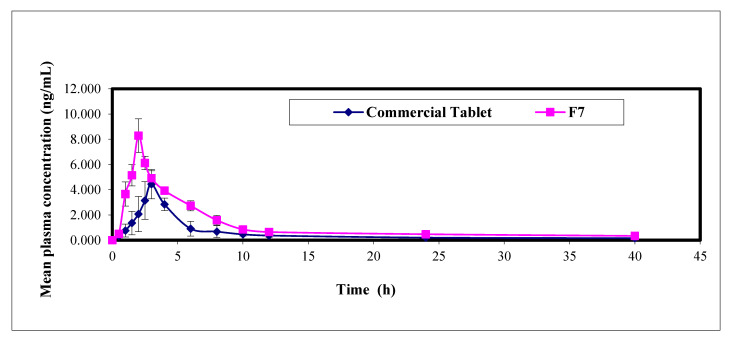
The mean plasma concentration time profiles of BH following iontophoretic transdermal delivery of F7 and single oral administration of Concor^®^ tablets to six human volunteers.

BH mean plasma concentration time profiles following transdermal iontophoretic delivery of F7 and administration of single oral dose of Concor^®^ tablets (5 mg) to six healthy human volunteers are shown in Figure 5. Related PK data are summarized in Table 5. The mean C_max_ determined from the selected hydrogel, was 1.9-fold higher and statistically significantly different (*p* = 0.0001) compared to Concor^®^ tablets. In addition, the mean determined T_max_ was smaller (2.00 ± 2.00 h) and statistically significantly different (*p* = 0.002) compared to the conventional tablets. The AUC_0–40_ values after transdermal and oral treatments were 43.19 ± 3.31 and 21.44 ± 3.60 ng·h/mL, respectively. Statistically significant difference was found between the two values (*p* = 0.0001), where the calculated relative bioavailability of BH after transdermal iontophoresis was about 201.44%, relative to Concor^®^ tablets. The significant higher values of both C_max_ and AUC_0–40_ together with the significant lower value of T_max_ compared to the conventional oral tablet confirm the enhanced transdermal transport of BH through combined action of permeation enhancers and iontophoresis. This could be due to the loosened structure of SC layers caused by their dual effect, which correlates well with the previous ex vivo permeation results. On the other hand, the mean elimination half-life (T_1/2_) and the mean residence time (MRT) were found to be insignificantly different (*p* > 0.05) for F7, compared to oral tablets. These results strongly cohere with the PK theory, where drug elimination is not affected by enhanced drug permeation and absorption. Similar findings were reported by Fouad et al. in their study on sublingual and intranasal delivery of dapoxetine hydrochloride versus commercial oral tablets [23].

Based on the previous findings, it can be concluded that F7 enhanced transdermal iontophoretic delivery of BH through the human’s skin. However, owing to the minute number of volunteers participating in the study, results can be preliminary. Therefore, further studies should be performed to prove the applicability of utilizing chemical enhancers combined with iontophoresis for transdermal permeation of BH.

## 10. Conclusions

To conclude, our study showed enhanced transdermal delivery of BH into human skin through the utility of combined strategy of both iontophoresis and permeation enhancers. These findings pointed to a successful alternative to conventional oral tablets which can, in return, solve the problem of dysphagia in elderly patients. Overcoming the hydrophilic nature of BH along with its high molecular weight via dual action of iontophoresis and permeation enhancers is the main reason for enhanced BH transdermal delivery. These results were confirmed via both ex vivo permeation and in vivo pharmacokinetic studies. Cellosolve^®^ hydrogel (F7) exhibited significant increase in transdermal permeation parameters, including Q_180_, Jss, and FER, through albino Wistar rat skin. It also showed significantly enhanced bioavailability compared to traditional Concor^®^ tablets in human volunteers. The methodology described in our study can be applied to other hydrophilic drug actives, in order to achieve alternative enhanced transdermal delivery. Further clinical studies should be performed, employing a larger number of human volunteers to prove the clinical efficacy of F7 in hypertension.

## Figures and Tables

**Table 1:** Composition of BH hydrogels.

Hydrogels *	Gelling Agents			Permeation Enhancers		
	Cp940	HEC	SCMC	Dermarol^TM^ 6CC	Cellosolve^®^	Tween^®^ 80
F1	1.00 g	-	-	-	-	-
F2	-	1.00 g	-	-	-	-
F3	-	-	1.00 g	-	-	-
F4	-	0.75 g	-	-	-	-
F5	-	0.50 g	-	-	-	-
F6	-	0.50 g	-	5.00 mL	-	-
F7	-	0.50 g	-	-	5.00 mL	-
F8	-	0.50 g	-	-	-	5.00 mL
F9	-	0.50 g	-	-	7.50 mL	-

* All formulations contained 0.2% *w*/*w* BH and 10% *w*/*w* propylene glycol.

**Table 2:** Physical appearance, drug content, and pH of hydrogels.

Hydrogels	Physical Appearance			Drug Content (%) (*n* = 3 ± SD)	pH (*n* = 3 ± SD)
	Color	Clarity	Homogeneity		
F1	Colorless	Turbid	Homogenous	98.00 ± 0.13	7.42 + 0.01
F2	Colorless	Clear	Homogenous	99.80 ± 0.94	7.44 + 0.03
F3	Colorless	Clear	Homogenous	101.43 ± 1.13	7.39 + 0.04
F4	Colorless	Clear	Homogenous	101.92 ± 0.93	7.42 + 0.02
F5	Colorless	Turbid	Homogenous	101.67 ± 1.18	7.41 + 0.01
F6	Colorless	Turbid	Homogenous	99.32 ± 0.72	7.38 + 0.04
F7	Colorless	Clear	Homogenous	99.57 ± 1.01	7.36 + 0.01
F8	Slightly yellow	Clear	Homogenous	101.51 ± 1.16	7.43 + 0.03
F9	colorless	Clear	Homogenous	103.00 ± 0.76	7.37 + 0.02

**Table 3:** Rheological properties of BH hydrogels.

Hydrogels	Viscosity at 50 rpm (cp)	Farrow’s Constant (N)	Flow Behavior
F1	2976	1.86	Pseudoplastic
F2	1027	1.72	Pseudoplastic
F3	681	1.12	Pseudoplastic
F4	645	2.46	Pseudoplastic
F5	3765	1.04	Pseudoplastic
F6	5361	1.21	Pseudoplastic
F7	675	2.58	Pseudoplastic
F8	978	3.20	Pseudoplastic
F9	981	1.12	Pseudoplastic

**Table 4:** Ex vivo permeation parameters of BH through rat skin.

Formulations *	Q_180_ (µg/cm^2^)	Q_240_ (µg/cm^2^)	Jss (µg·h/cm^2^)	KP (cm/h)	FER
BH solution by passive diffusion	5.24 ± 0.06	5.77 ± 0.05	1.44 ± 0.01	0	1
BH solution by iontophoresis	371.68 ± 3.78	415.92 ± 4.44	103.98 ± 1.11	0.01040 ± 0.00011	72.09 ± 0.69
F1	652.00 ± 8.06	719.43 ± 0.62	179.86 ± 0.16	0.01799 ± 0.00002	124.70 ± 1.03
F2	706.41 ± 5.14	792.38 ± 9.43	198.10 ± 2.36	0.01981 ± 0.00024	137.34 ± 1.12
F3	598.99 ± 5.24	685.12 ± 6.56	171.28 ± 1.64	0.01713 ± 0.00016	118.75 ± 1.35
F4	742.04 ± 5.67	850.49 ± 5.35	212.62 ± 1.34	0.02126 ± 0.00013	147.42 ± 1.22
F5	900.57 ± 8.79	982.61 ± 7.04	245.65 ± 1.76	0.02457 ± 0.00018	170.31 ± 0.33
F6	1058.78 ± 18.37	1146.67 ± 5.03	286.67 ± 1.26	0.02867 ± 0.00013	198.75 ± 1.12
F7	1390.68 ± 3.65	1470.84 ± 9.49	367.71 ± 2.37	0.03677 ± 0.00024	254.94 ± 1.32
F8	1186.55 ± 12.00	1292.02 ± 6.67	323.00 ± 1.67	0.03230 ± 0.00017	223.94 ± 1.25
F9	1307.44 ± 4.26	1415.93 ± 4.70	353.98 ± 1.18	0.03540 ± 0.00012	245.43 ± 2.65

* All values are represented as mean data (*n* = 3 ± SD).

**Table 5:** Mean pharmacokinetic parameters of BH following transdermal iontophoretic permeation of F7 and oral administration of Concor^®^ tablets to six human volunteers.

PK Parameters *	F7	Concor^®^ Tablet	Statistical *p*-Values
C_max_ (ng/mL)	8.28 ± 1.34	4.46 ± 1.13	*p* = 0.0001
T_max_ (h) ^a^	2.00 ± 2.00	2.92 ± 3.00	*p* = 0.0020
AUC_0–40_ (ng·h/mL)	43.19 ± 3.31	21.44 ± 3.60	*p* = 0.0001
MRT (h)	8.69 ± 1.86	8.59 ± 2.77	*p* > 0.0500

* Data are mean values (*n* = 6 ± SD), ^a^ Median.
